# A combination approach to treating fungal infections

**DOI:** 10.1038/srep17070

**Published:** 2015-11-23

**Authors:** Sanjib K. Shrestha, Marina Y. Fosso, Sylvie Garneau-Tsodikova

**Affiliations:** 1 Department of Pharmaceutical Sciences, University of Kentucky, Lexington, KY, 40536-0596, USA

## Abstract

Azoles are antifungal drugs used to treat fungal infections such as candidiasis in humans. Their extensive use has led to the emergence of drug resistance, complicating antifungal therapy for yeast infections in critically ill patients. Combination therapy has become popular in clinical practice as a potential strategy to fight resistant fungal isolates. Recently, amphiphilic tobramycin analogues, **C**_**12**_ and **C**_**14**_, were shown to display antifungal activities. Herein, the antifungal synergy of **C**_**12**_ and **C**_**14**_ with four azoles, fluconazole (FLC), itraconazole (ITC), posaconazole (POS), and voriconazole (VOR), was examined against seven *Candida albicans* strains. All tested strains were synergistically inhibited by **C**_**12**_ when combined with azoles, with the exception of *C. albicans* 64124 and MYA-2876 by FLC and VOR. Likewise, when combined with POS and ITC, **C**_**14**_ exhibited synergistic growth inhibition of all *C. albicans* strains, except *C. albicans* MYA-2876 by ITC. The combinations of FLC-**C**_**14**_ and VOR-**C**_**14**_ showed synergistic antifungal effect against three *C. albicans* and four *C. albicans* strains, respectively. Finally, synergism between **C**_**12**_/**C**_**14**_ and POS were confirmed by time-kill and disk diffusion assays. These results suggest the possibility of combining **C**_**12**_ or **C**_**14**_ with azoles to treat invasive fungal infections at lower administration doses or with a higher efficiency.

Invasive fungal infections such as candidiasis have become a major cause of mortality and morbidity, especially among immunocompromised (HIV, cancers) and critically ill patients worldwide^1^,^2^. The National Healthcare Safety Network (NHSN) at the Centers of Diseases Control and Prevention (CDC) has reported that *Candida* spp. ranked the fifth among hospital-acquired pathogens^3^. *Candida* spp. have also been reported as the fourth most common causative pathogens of nosocomial bloodstream infection claiming more lives in the United States^4^.

Azoles, echinocandins, allylamines, and polyenes are the four major classes of antifungal agents that are used to treat candidiasis as well as other type of fungal infections in humans. Among these four, azoles such as fluconazole (FLC), itraconazole (ITC), posaconazole (POS), and voriconazole (VOR) are considered first line drugs to treat refractory fungal diseases (Fig. 1)^5^. The fungistatic nature and prolonged use of azoles to treat fungal infections, however, has promoted the selection and emergence of drug resistant fungal strains. This necessitates either the development of novel antifungal drugs or improved therapeutic strategy to overcome drug resistance problems by *C. albicans*. In clinical settings, combination therapy has become a potential alternative to treat invasive fungal infections by improving clinical efficacy of existing drugs such as azoles and reducing their side effects to host by lowering administrative doses. Previously, promising results were observed by combining azoles with different compounds such as tacrolimus (FK506)^6^, cyclosporine A^7^, amiodarone^8^, and retigeric acid B^9^ against *C. albicans* strains.

We recently demonstrated that modifying the aminoglycoside tobramycin (TOB) at the 6”-position by incorporating linear alkyl chains (C_6_–C_22_) in a thioether linkage resulted in chain-length-dependent antibacterial and antifungal activities against various bacteria and fungi with resistance to the parent drug, TOB, itself^10^,^11^. This was especially true for TOB derivatized with linear alkyl chains of 12 and 14 carbons in length (referred to as **C**_**12**_ and **C**_**14**_ from here on) (Fig. 1). However, synergistic interactions between the amphiphilic aminoglycosides **C**_**12**_ and **C**_**14**_ and azoles against fungal strains have not yet been explored. In this study, in an effort to establish if TOB derivatives could be used in combination with currently available antifungal agents, we evaluated the combined effects of **C**_**12**_ and **C**_**14**_ with four azoles against azole-sensitive and azole-resistant *C. albicans* by checkerboard, time-kill curve, and disk diffusion assays. Additionally we have determined the *in vitro* cytotoxicity effect of TOB analogues and azoles in combination against mammalian cells.

## Results

### In vitro antifungal activities of drugs alone

Prior to investigating the effect of combining **C**_**12**_ or **C**_**14**_ with four azoles (FLC, ITC, POS, and VOR), the MIC values of these compounds were determined individually against seven strains of *C. albicans* (Tables 1 and 2). The clinical sources and susceptibility/resistance profile of these strains, as reported by the American Type Culture Collection (ATCC), are presented in Table S1.

Based on complete inhibition (MIC-0) (Tables 1 and 2), **C**_**12**_ and **C**_**14**_ displayed MIC values of 16–32 μg/mL and 8 μg/mL, respectively, against all *C. albicans* strains tested. These MIC values are consistent with those previously reported for these compounds against these specific yeast strains^11^. When compared to **C**_**12**_ and **C**_**14**_, FLC, ITC, POS, and VOR displayed higher MIC values against the majority of the yeast strains tested (MIC values ranged from ≥25 μg/mL, 12.5- >25 μg/mL, 10- >20 μg/mL, and ≥10 μg/mL, respectively), with the exception of the *C. albicans* ATCC 10231 (**A**) strain where ITC, POS, and VOR had MIC values of 0.78 μg/mL, 0.62 μg/mL, and 0.31 μg/mL, respectively. The MIC values of azoles against yeast strains were determined based on 50% inhibition or MIC-0 and were consistent with previously reported MICs for these compounds. However, due to the long trailing growth effects by azole susceptible strains *C. albicans* MYA-2876 (**C**) and *C. albicans* MYA-2310 (**E**), we did observe higher MIC-2 values for all azoles against these strains. To validate our MIC data of azoles against these two strains (**C** and **E**), we also tested caspofungin as a control in the same set of MIC testing experiments. It is important to note that ATCC has reported these two strains (**C** and **E**) as sensitive to caspofungin. Unlike azoles, caspofungin showed complete inhibition at <0.48 μg/mL against these strains (Table S2).

### **In vitro** synergistic antifungal activities

Having established the individual MIC values for **C**_**12**_, **C**_**14**_, FLC, ITC, POS, and VOR, the MIC and FICI values of **C**_**12**_ and **C**_**14**_ in combination with the four azoles (FLC, ITC, POS and VOR) were determined in checkerboard assays against the seven strains of *C. albicans* (Tables 1 and 2). When combined with FLC or ITC or POS or VOR, **C**_**12**_ showed strong synergistic inhibitory effects against the majority of the *C. albicans* strains tested with FICI values ranging from 0.07–0.5 (FLC or ITC or POS plus **C**_**12**_) and 0.07–0.27 (VOR plus **C**_**12**_). The only combinations for which no synergistic effects were observed were FLC plus **C**_**12**_ (FICIs = 0.51 and 1) or VOR plus **C**_**12**_ (FICIs = 0.62 and 0.75) against *C. albicans* ATCC 64124 (**B**) and *C. albicans* ATCC MYA-2876 (**C**), respectively. Likewise, the combination of **C**_**14**_ with FLC or ITC or POS or VOR also exhibited good synergy against the majority of the *C. albicans* strains tested, with FICI values ranging from 0.28–0.5 (FLC plus **C**_**14**_), 0.18-0.5 (ITC plus **C**_**14**_), 0.18-0.49 (POS plus **C**_**14**_), and 0.14–0.37 (VOR plus **C**_**14**_). With **C**_**14**_, the combinations for which no synergistic effects were observed were FLC or VOR plus **C**_**14**_ against *C. albicans* ATCC 10231 (**A**), *C. albicans* ATCC 64124 (**B**), and *C. albicans* ATCC MYA-2876 (**C**), as well as FLC plus **C**_**14**_ (FICI = 1) against *C. albicans* ATCC MYA-1003 (**G**).

### Time-kill studies of drug combinations

**Figure 2:**
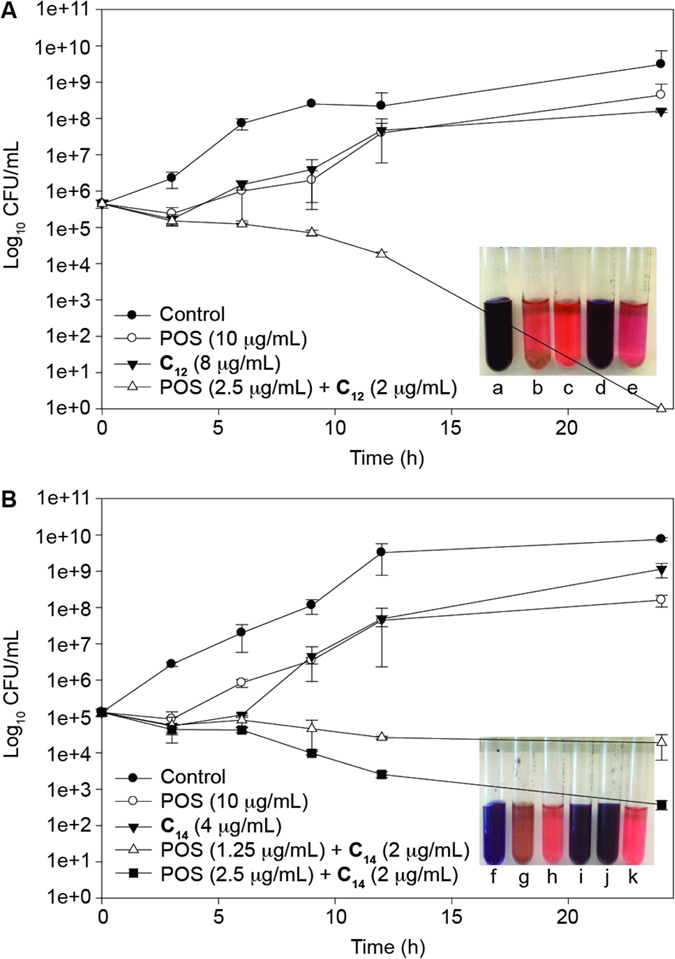
Representative time-kill studies of 6”-thioether TOB analogues **C**_**12**_ (panel **A**) or **C**_**14**_ (panel **B**) with POS alone and in combination against azole-resistant *C. albicans* ATCC 64124 (strain **B**). (**A**) Cultures were exposed to **C**_**12**_ at 8 μg/mL (black inverted triangle), POS at 10 μg/mL (white circle), the combination of **C**_**12**_ at 2 μg/mL and POS at 2.5 μg/mL (white triangle), and no drug (control, black circle). (**B**) Cultures were exposed to **C**_**14**_ at 4 μg/mL (black inverted triangle), POS at 10 μg/mL (white circle), and the combination of **C**_**14**_ at 2 μg/mL and POS at 1.25 μg/mL (white triangle) or **C**_**14**_ at 2 μg/mL and POS at 2.5 μg/mL (black square), and no drug (control, black circle). *Note*: inset in panels (**A**,**B**) After 24 h of no drug/drug exposure, cultures of *C. albicans* ATCC 64124 (strain **B**) were further treated with Alamar Blue dye (25 μg/mL) and incubated at room temperature in the dark for another 10  h. Culture tubes showing red indicates cell survival whereas blue indicates cell death. Lanes a and f = sterility control; b and g = growth control; c and h = POS (10 μg/mL); d, i, and j = POS + AG derivative (concentrations are POS (2.5 μg/mL) + **C**_**12**_ (2 μg/mL) or POS (1.25 μg/mL) + **C**_**14**_ (2 μg/mL) or POS (2.5 μg/mL) + **C**_**14**_ (2 μg/mL)); e and k = AG derivative alone (**C**_**12**_ (8 μg/mL) or **C**_**14**_ (4 μg/mL)).

To confirm the synergistic inhibitory effects of **C**_**12**_ or **C**_**14**_ and POS against the azole-resistant *C. albicans* ATCC 64124 (**B**) strain, representative time-kill studies were performed (Fig. 2). At 8 or 4 μg/mL, **C**_**12**_ or **C**_**14**_ alone did not show inhibition to the growth of *C. albicans* ATCC 64124 (**B**). In contrast, POS, at 10 μg/mL, showed inhibition for the first 3 h of growth of the yeast strain, and after that the growth was similar to that of the growth control (no drug). However, the combined administration of **C**_**12**_ (2 μg/mL) with POS (1.25 μg/mL) and **C**_**14**_ (2 μg/mL) with POS (1.25 or 2.5 μg/mL) against *C. albicans* ATCC 64124 (**B**) yielded a ≥2 log_10_ decrease in CFU/mL after 9 h and 12 h of treatment compared with each compound alone, respectively (Fig. 2). The results obtained by time-kill studies are consistent with those from the checkerboard assays.

### Disk diffusion assays

**Figure 3:**
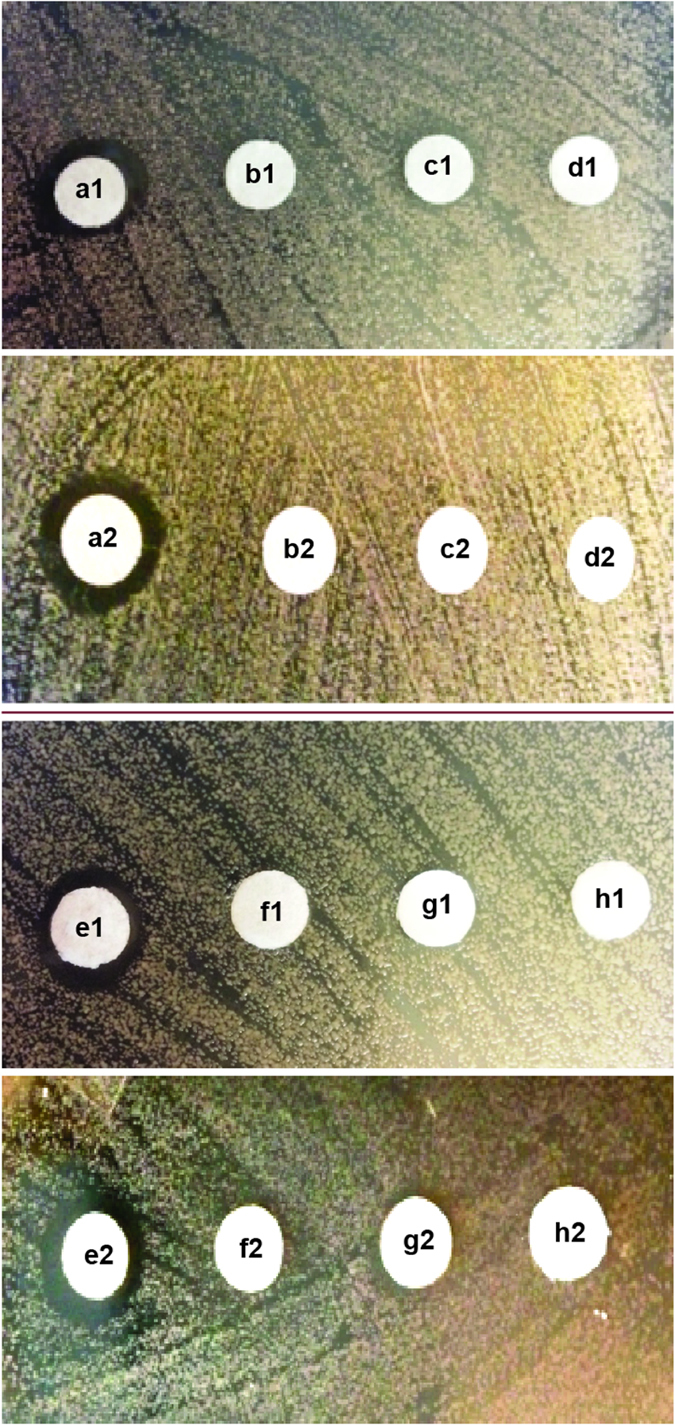
figtlDisk diffusion assay (done in duplicate; series 1 and 2) showing that C_14_, when used in combination with the azoles POS or ITC, kills *C. albicans* ATCC 64124 (strain B). *Note*: at the concentrations tested, **C**_**14**_, POS, or ITC do not kill the fungal strain. **a1** and **a2** = POS (100 μg) + **C**_**14**_ (500 μg); **b1** and **b2** = POS (100 μg); **c1** and **c2** = **C**_**14**_ (500 μg); **d1** and **d2** = H_2_O; **e1** and **e2** = ITC (150 μg) + **C**_**14**_ (700 μg); **f1** and **f2** = ITC (150 μg); **g1** and **g2** = **C**_**14**_ (700 μg); **h** = H_2_O.

To examine the nature of the drug interactions between **C**_**14**_ with POS or ITC against the azole-resistant *C. albicans* ATCC 64124 (**B**) strain, disk diffusion assays were performed in duplicate. **C**_**14**_ (500 or 700 μg/mL), POS (100 μg/mL), and ITC (150 μg/mL) alone, when applied on disk, did not show a zone of inhibition against *C. albicans* ATCC 64124 (**B**). However, when co-spotted, **C**_**14**_ (500 μg/mL) and POS (100 μg/mL) or **C**_**14**_ (700 μg/mL) and ITC (150 μg/mL) resulted in a visible zone of inhibition against this strain, which confirmed the synergistic antifungal interactions of these compounds (Fig. 3).

### Cytotoxic effect of drug combinations

**Figure 4:**
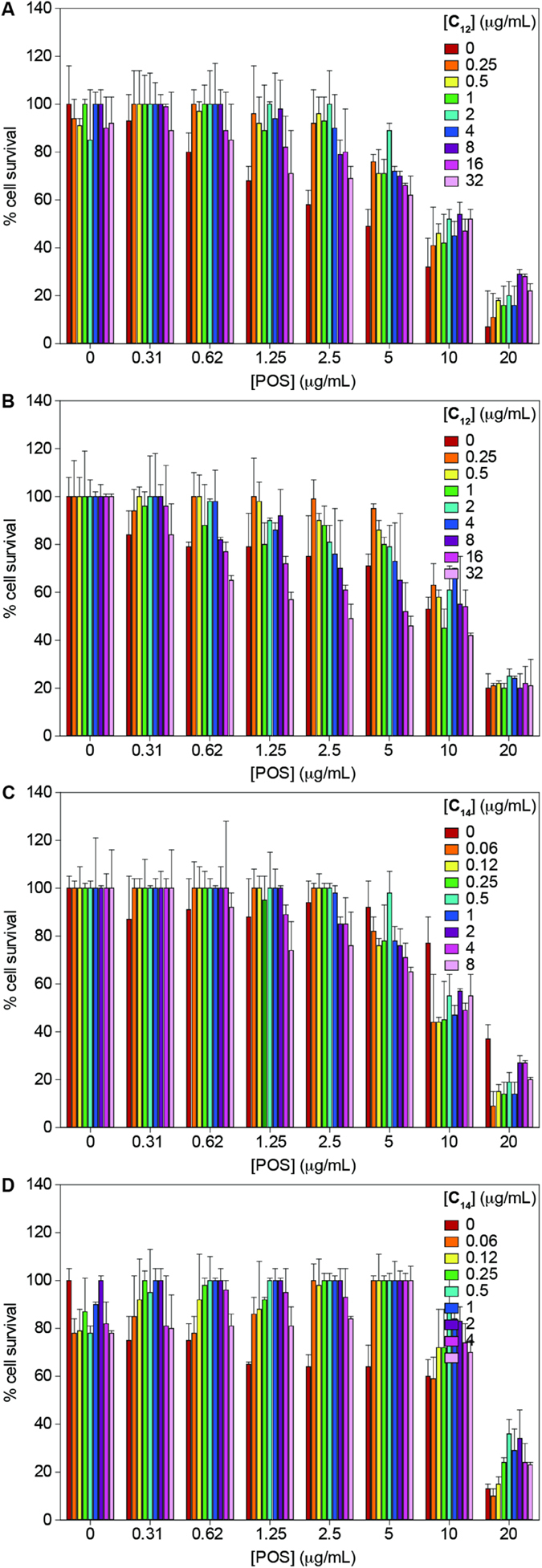
Cytotoxicity of 6”-thioether **C**_**12**_ or **C**_**14**_ TOB analogues and POS alone and in combination against mammalian cells. (**A**,**C**) A549 cells and (**B**,**D**) BEAS-2B cells were treated with **C**_**12**_ or **C**_**14**_, and POS alone and in combinations at various concentrations and incubated at 37 °C for 24 h in a CO_2_ incubator.

To investigate the cytotoxic effects of **C**_**12**_ or **C**_**14**_ and POS alone and in combinations, assays were performed against A549 and BEAS-2B cells (Fig. 4 and Tables S3-S6). As we previously reported^11^, **C**_**12**_ or **C**_**14**_, at their respective highest antifungal MIC values of 32 μg/mL and 8 μg/mL, basically did not show toxicity against the A549 and BEAS-2B cell lines. On the other hand, the newly tested POS at 20  μg/mL, which is a concentration below its antifungal MIC value against *C. albicans* ATCC 64124 (**B**), exhibited severe toxicity against the A549 and BEAS-2B cell lines, resulting in ≤37% cell survival in both cases. On a very positive note, when tested at 8-fold higher concentrations of POS (10 μg/mL) plus **C**_**12**_ or **C**_**14**_ (16 or 8 μg/mL) in combinations than their synergistic antifungal MIC values (*Note*: the synergistic MIC values for POS and **C**_**12**_, or **C**_**14**_ in combinations are 1.25 and 2 or 1), only minimal or no toxicity were observed against the A549 and BEAS-2B cell lines, resulting in ≥47% cell survival in both cases.

## Discussion

Opportunistic fungal infections have become a serious threat to human health due to the rising population of immunocompromised patients as result of HIV infections, chemotherapy, and organ transplant^12^. Azoles are drugs of choice for antifungal therapy for various fungal infections in humans, including candidiasis. However drug-drug interactions, severe side effects, and development of resistance have limited their therapeutic efficacies against fungi^13^. Thus, new strategies are warranted to overcome antifungal drug resistance and side effects due to use of high doses of these drugs.

In this study, we investigated the *in vitro* antifungal synergy of two amphiphilic TOB derivatives, **C**_**12**_ and **C**_**14**_, with four azoles (FLC, ITC, POS and VOR) against seven azole-resistant and azole-sensitive strains of *C. albicans*. Our results demonstrated that **C**_**12**_ and **C**_**14**_ exhibit potent antifungal synergy *in vitro* with all four azoles against the majority of the *C. albicans* strains tested. Despite displaying less antifungal activity when used alone, **C**_**12**_ alone (16–32 μg/mL) compared to **C**_**14**_ alone (8 μg/mL), **C**_**12**_demonstrated better synergistic inhibitory effects when combined with azoles against all strains of *C. albicans* tested with FICI values ranging from 0.07–0.5. The only combinations for which no synergy was detected were those of **C**_**12**_ and FLC or VOR against *C. albicans* ATCC 64124 (**B**) and *C. albicans* ATCC MYA-2876 (**C**) (Table 1). Similarly, **C**_**14**_ also did not display synergy when used in combination with FLC and VOR against these strains. Although **C**_**14**_ also displayed good antifungal synergy in combinations with all azoles against the majority of the fungal strains tested (FICI values ranging from 0.14–0.5), more combinations yielded no synergy. In addition to the **C**_**14**_ with FLC or VOR against strains **B** and **C**, indifference was observed with the combinations of FLC or VOR with **C**_**14**_ against *C. albicans* ATCC 10231 (**A**), FLC with **C**_**14**_ against *C. albicans* ATCC MYA-1003 (**G**), as well as ITC with **C**_**14**_ against *C. albicans* ATCC MYA-2876 (**C**) (Table 2). It is also noteworthy to mention that the MIC values of all azoles were greatly reduced in presence of **C**_**12**_or **C**_**14**_ against various fungal strains. For example, the MIC values of POS were reduced by 64-fold against *C. albicans* ATCC 90819 (**D**) in the presence of **C**_**12**_or **C**_**14**_. Also, POS lowered the MIC values of **C**_**12**_ or **C**_**14**_ by 4-fold against same strain in both case. Alternative methods, such as time-kill studies and disk diffusion assays, were also performed to evaluate the drug interactions of **C**_**12**_ and **C**_**14**_ with POS or ITC (used for disk diffusion assays only) against *C. albicans* ATCC 64124 (**B**). The results obtained further confirmed the synergistic interactions of **C**_**12**_and **C**_**14**_ with POS and were in agreement with the results obtained by checkerboard analysis against specific yeast strains. Interestingly, although we did observe zones of inhibition for **C**_**12**_ and **C**_**14**_ with POS or ITC against *C. albicans* ATCC 64124 (**B**) in our disk diffusion assay, these were small. Probably, the higher molecular weight of TOB analogues may have contributed the poor diffusion of these compounds through agar^14^ or interaction of these polycationic compounds with sulfates and acids of agar polymer may have resulted reduced inhibition with minor zone of inhibition^15^. Interestingly, when tested with antifungals other than azoles such as caspofungin (an echinocandin) and naftifine (an allylamine), **C**_**12**_ and **C**_**14**_ did not show synergy, at least against one strain of *C. albicans*, *C. albicans* ATCC 64124 (**B**) (data not shown).

In this study, we included clinical isolates of *C. albicans* strains that are reported as azole (FLC, ITC and VOR) resistant strains, except for two strains, *C. albicans* ATCC MYA-2876 (**C**) and *C. albicans* ATCC MYA-2310 (**E**), which are reported as azole-sensitive. In the majority of cases, **C**_**12**_and **C**_**14**_ exhibited synergistic inhibitory effects with azoles against these strains. These observations indicates that combination therapy using **C**_**12**_or **C**_**14**_ with an azole may provide a new strategy to fight fungal infections caused by resistant strains like *C. albicans* ATCC 64124 (**B**) that has mutations in its *ERG11* sequences^16^,^17^.

Having established the synergistic antifungal interactions of **C**_**12**_and **C**_**14**_ with azoles, and knowing their non-cytotoxicity effects against A549 and BEAS-2B mammalian cell lines^11^, we further evaluated the cytotoxicity effects of **C**_**12**_and **C**_**14**_ in combination with POS against the A549 and BEAS-2B cell lines. At above 8-fold higher than or equal to their synergistic antifungal MIC values, **C**_**12**_and **C**_**14**_ with POS exhibited minimal to no toxicity against these cell lines resulting in ≤47% cell survival (Fig. 4 and Tables S3-S6). These results may suggest that the clinical efficacies of azoles can be resumed by achieving low doses with less toxicity when combined with **C**_**12**_or **C**_**14**_ to treat stubborn mycoses. Besides, the results may provide flexibility to extrapolate the range of concentrations that can be used in combination to perform *in vivo* experiments.

Certain amphiphilic aminoglycosides such as FG08 and K20 were reported to inhibit fungi by disrupting fungal membrane^18^,^19^,^20^. Recently, we reported that **C**_**12**_and **C**_**14**_ inhibit fungi by inducing apoptosis leading to fungal membrane disruption^11^. On the other hand, azoles kill fungi by inhibiting the cytochrome P450-dependent enzyme sterol 14-α-demethylase involved in ergosterol biosynthesis. The mechanism by which **C**_**12**_ and **C**_**14**_ synergize with azoles remains to be established in studies that are out of scope for this manuscript. One of the major mechanism of resistance to azoles by fungi is due to up-regulation of efflux pumps (CDR1 and CDR2) that lower the intracellular drug concentrations^21^. When azoles are combined with **C**_**12**_ and **C**_**14**_, it could be expected that **C**_**12**_ and **C**_**14**_ could enhance azoles permeability to fungi by altering fungal membrane integrity that may intensify the fungal killing. However, the cascades of multiple secondary effects such as reactive oxygen species (ROS) accumulation, mitochondrial membrane potential dissipation, and DNA condensation and fragmentation as a result of membrane disruption action cannot be overlooked as a cause of death^22^.

## Conclusions

In conclusion, our study demonstrated the synergistic combination effects between **C**_**12**_or **C**_**14**_ and four azoles against the majority of the *C. albicans* strains tested. These synergistic interactions were further confirmed by time-kill curves and disk diffusion assays. The combination effects of **C**_**12**_or **C**_**14**_ and azoles appears non toxic to mammalian cells at higher or equal to synergistic antifungal MIC values of these drugs against fungi. **C**_**12**_ or **C**_**14**_-azoles combination therapy might be mainly beneficial to treat invasive fungal infections like candidiasis. Future studies in our laboratory will be focused on establishing the mechanism of action of these drugs in combination.

## Materials and Methods

### Materials

Tobramycin (TOB) was purchased from AK scientific (Union City, CA). All other chemicals were purchased from Sigma Aldrich (St. Louis, MO) and used without further purification. TOB analogues with linear alkyl chains **C**_**12**_ and **C**_**14**_ were synthesized as described previously^10^ and were dissolved in double distilled water (ddH_2_O) at a final concentration of 10 g/L for storage at −20 °C.

### Antifungal agents

The antifungal agents fluconazole (FLC), itraconazole (ITC), posaconazole (POS), and voriconazole (VOR) were obtained from AK Scientific, Inc. (Mountain View, CA). FLC, ITC, POS, and VOR were dissolved in DMSO at a final concentration of 5 g/L. All of these solutions were stored at −20 °C.

### Fungal strains and culture conditions

The yeast strains *C. albicans* ATCC 10231 (**A**), *C. albicans* ATCC 64124 (**B**), and *C. albicans* ATCC MYA-2876 (**C**) were kindly provided by Dr. Jon Y. Takemoto (Utah State University, Logan, UT, USA). The yeast strains *C. albicans* ATCC 90819 (**D**), *C. albicans* ATCC MYA-2310 (**E**), *C. albicans* ATCC MYA-1237 (**F**), and *C. albicans* ATCC MYA-1003 (**G**) were purchased from the ATCC (Manassas, VA, USA). All yeast strains were cultivated at 35 °C in RPMI 1640 medium.

### *In vitro* antifungal activities

Based on the previously reported MIC values for **C**_**12**_, **C**_**14**_, FLC, ITC, POS^11^, appropriate ranges of concentrations for *in vitro* drug combination studies were determined. It is important to note that the MIC values for **C**_**12**_, **C**_**14**_, FLC, ITC, POS alone were again determined here to allow for direct comparison with combination results. In the current study, MIC values for **C**_**12**_, **C**_**14**_, FLC, ITC, POS, and VOR against different fungal strains were determined as described in the CLSI document M27-A3^23^ with minor modifications. Some of our fungal strains, such as *C. albicans* ATCC 64124 (strain **B**), tend to produce pseudohyphae (filaments) in RPMI 1640 medium, which has been found to compromise cell counting when using a hemocytometer. Therefore, we used potato dextrose broth (PDB) to prepare yeast inocula and later diluted in RPMI 1640 medium to perform MIC value determination, as well as checkerboard and time-kill assays. Modifications included growing yeast cells in potato dextrose broth (PDB) for 24–48 h at 35 °C, diluting the yeast culture in RPMI 1640 medium to a concentration of 1 × 10^6^ cells/mL (as determined by using an hemocytometer) and using a final inoculum size of 5 × 10^4^ cfu/mL for all the assays (*Note*: identical results were obtained when using 5 × 10^3^ cfu/mL and 5 × 10^4^ cfu/mL as a final inoculum size when tested against strain **B**. As it is known that a higher inoculum size of cells can raise the MIC values determined, we selected 5 × 10^4^ cfu/mL to provide conditions that would lead to the highest MIC values possible for our compounds so that we could really determined their potential). Two-fold serial dilution of **C**_**12**_, **C**_**14**_, FLC, ITC, POS, and VOR was prepared using RPMI 1640 medium (100 μL) and cell suspension (100 μL) was added to 96-well microtiter plate to achieve final drug and inoculum concentration of 0.15–10 mg/L and 5 × 10^4^ cfu/mL, respectively. Plates were incubated for 48 h at 35 °C. The MIC values for all azoles studied were defined as the lowest drug concentration that inhibits 50% of fungal cell growth or MIC-2. The MIC values for **C**_**12**_ and **C**_**14**_ were defined as the lowest drug concentration that yielded complete growth inhibition or MIC-0.

### Determination of percentage of yeast cell growth inhibition used to determine MIC-2 values for FLC, ITC, POS, VOR, and caspofungin

To confirm the susceptibility profile of yeast strains **C** and **E**, we determined the percentage of *C. albicans* ATCC MYA-2876 (**C**) and *C. albicans* ATCC MYA2310 (**E**) growth inhibition by FLC, ITC, POS, VOR, and caspofungin. The experiments were performed as described above for the *in vitro* antifungal activities and percentages of growth at concentrations varying from 0.48-31.25 μg/mL of azoles or caspofungin were measured by reading absorbance at 600 nm (A_600_) using a SpectraMax M5 plate reader.

### Antifungal checkerboard analysis

The synergistic interaction between **C**_**12**_ and **C**_**14**_ with four azoles (FLC, ITC, POS, and VOR) was evaluated against various strains of *C. albicans* using a microdilution checkerboard assay according to CLSI M27-A3^23^. The test was performed in 96-well plates using RPMI 1640 medium. It is important to note that the MIC values were also determined for all azoles and TOB analogues alone in the same set of experiments in checkerboard assays for comparision. These MIC values are not from previous reports. The final concentration of yeast cells used was 1 × 10^4^ cfu/mL as verified by colony counting. The final concentration of drugs ranged from 0.25–32 μg/mL for **C**_**12**_, 0.06–8 μg/mL for **C**_**14**_, 0.39–25 μg/mL for FLC, 0.39–25 μg/mL for ITC, 0.31–20 μg/mL for POS, and 0.31–10 μg/mL for VOR. Plates were incubated for 48  h at 35 °C. Each test was performed in duplicate. A non-parametric model based on Loewe Additivity (LA) theory was used to analyze the nature of *in vitro* interaction of **C**_**12**_ and **C**_**14**_, and all four azoles using fractional inhibitory concentration index (FICI)^24^. According to LA theory, FICI can be defined as the sum of the ratios of the MIC values of each drug when used in combination to their respective MIC values when used alone. Drug interactions were classified as synergistic (SYN), indifferent (IND), or antagonistic (ANT) according to the fractional inhibitory concentration index (FICI). The interaction was defined as synergistic if the FICI was ≤0.5, indifferent if >0.5–4, and antagonistic if >4.

### Time-kill studies of drug combinations

Representative time-kill studies were performed to investigate the activity of **C**_**12**_ and **C**_**14**_ in the presence or absence of POS against one azole-resistant strain, *C. albicans* ATCC 64124 (**B**). These assays were performed in 15 mL culture tubes using RPMI 1640 medium as previously described^25^. Different sets of cell suspensions were prepared with **C**_**12**_ (8 μg/mL), **C**_**14**_ (4 μg/mL), and POS alone (10 μg/mL), or combinations of **C**_**12**_ (2 μg/mL) plus POS (2.5 μg/mL) or **C**_**14**_ (2 μg/mL) plus POS (1.25 and 2.5 μg/mL), or growth control (no drug) and sterility control (no cells and no drug). The final inoculum size of yeast cells used was 10^5^ cfu/mL as confirmed by colony count. The cell suspensions were then incubated at 35 °C with constant shaking (200 rpm). Aliquot of 100 μL from each tubes were removed at 0, 3, 6, 9, 12, and 24 h, and serially diluted in sterile ddH_2_O. 50 μL of each dilution was plated onto potato dextrose agar (PDA) and then incubated at 35 °C. Colony counts were determined after 48 h of incubation. The experiments were performed in duplicate.

### Disk diffusion assays

The disk diffusion assays were performed in duplicate according to the CLSI document M44-A2^26^. *C. albicans* ATCC 64124 (**B**), at a density of ~5 × 10^5^ cfu/mL, were spread onto PDA plates. Sterile filter disks (~0.6 cm) were placed on the agar surface. 10 μL aliquot of POS (100 μg/mL), ITC (150 μg/mL), and **C**_**14**_ alone (either 500 or 700 μg/mL), or combinations of **C**_**14**_ (500 μg/mL) plus POS (100 μg/mL) or **C**_**14**_ (700 μg/mL) plus ITC (150 μg/mL) were loaded onto the disks and then incubated at 35 °C for 48 h before analysis.

### Cytotoxic effect of drug combinations

Cytotoxicity assays were performed as previously described^27^ with minor modifications. The human lung carcinoma epithelial cells A549 and the normal human bronchial epithelial cells BEAS-2B were grown in DMEM containing 10% fetal bovine serum (FBS) and 1% antibiotics. The confluent cells were then trypsinized with 0.05%-trypsin-0.53 mM EDTA and resuspended in fresh medium (DMEM). The cells were transferred into 96-well microtiter plates at a density of 3000 cells/well and were grown overnight. The following day, checkerboard plates were prepared to evaluate the cytotoxic effects of POS, and **C**_**12**_ or **C**_**14**_ alone and in combination against A549 and BEAS-2B cells. The checkerboard plates were prepared in a new 96-well microtiter plates as described above in antifungal checkerboard analysis except that drugs were diluted in DMEM medium in a final volume of 200 μL. The final concentration of drugs ranged from 0.25–32 μg/mL for **C**_**12**_, 0.06-8 μg/mL for **C**_**14**_, and 0.31–20 μg/mL for POS. The media containing cells were then replaced by 200 μL of fresh culture media containing drugs either alone or in combinations from the checkerboard plates. The cells were incubated for additional 24 h at 37 °C with 5% CO_2_ in a humidified incubator. To evaluate cell survival, each well was treated with 10 μL (25 mg/L) of resazurin sodium salt (Sigma-Aldrich) for 3–6 h. Metabolically active cells can convert the blue non-fluorescent dye resazurin to the pink and highly fluorescent dye resorufin, which can be detected at A_560_ excitation and A_590_ emission wavelengths by using a SpectraMax M5 plate reader. Triton X-100^®^ (1%, *v*/*v*) gave complete loss of cell viability and was used as the positive control. Percent cell survival was calculated as: (control value – test value)×100/control value, where control value represents cells + resazurin – drug, and test value represents cells + resazurin + drug.

## Additional Information

**How to cite this article**: Shrestha, S. K. *et al.* A combination approach to treating fungal infections. *Sci. Rep.* **5**, 17070; doi: 10.1038/srep17070 (2015).

## Supplementary Material

Supplementary Information

## Figures and Tables

**Figure 1:**
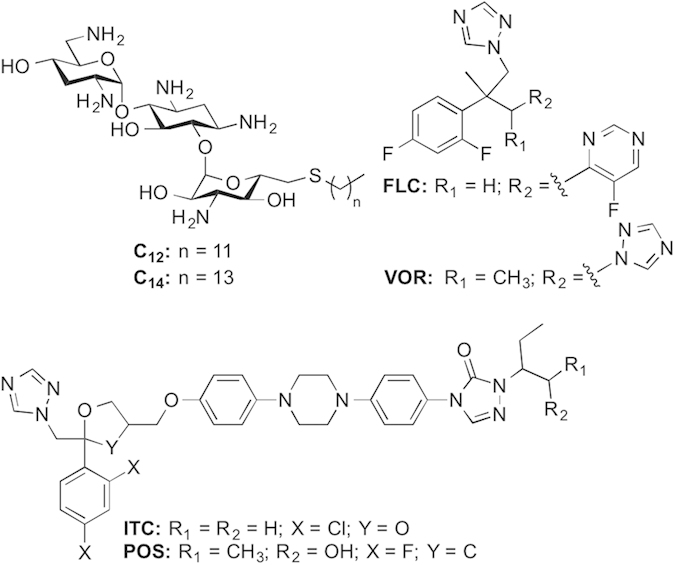
Structures of the 6”-thioether TOB analogues C_12_ and C_14_ and of the azole antifungal agents used in this combination study.

**Table 1:** *In vitro* susceptibility of yeast strains to C_12_ and azoles alone and in combination.

MICs of drugs (μg/mL)						
Drugs and Strains^a^	Alone		In combination		FICIs	Interpretation
	Azole	C_12_	Azole	C_12_		
FLC						
*C. albicans* 10231 (**A**)^b^	25	32	1.56	8	0.31	SYN
*C. albicans* 64124 (**B**)^b^	>25	32	0.78	16	0.53	IND
*C. albicans* MYA-2876 (**C**)^c^	>25	16	12.5	8	1	IND
*C. albicans* 90819 (**D**)^b^	>25	32	1.56	2	0.12	SYN
*C. albicans* MYA-2310 (**E**)^c^	>25	16	0.39	1	0.07	SYN
*C. albicans* MYA-1237 (**F**)^b^	>25	32	1.56	2	0.12	SYN
*C. albicans* MYA-1003 (**G**)^b^	>25	32	6.25	8	0.5	SYN
ITC						
*C. albicans* 10231 (**A**)^b^	0.78	32	0.049	4	0.18	SYN
*C. albicans* 64124 (**B**)^b^	>25	32	1.56	8	0.31	SYN
*C. albicans* MYA-2876 (**C**)^c^	12.5	16	3.12	4	0.5	SYN
*C. albicans* 90819 (**D**)^b^	>25	32	0.78	4	0.15	SYN
*C. albicans* MYA-2310 (**E**)^c^	12.5	16	0.39	2	0.15	SYN
*C. albicans* MYA-1237 (**F**)^b^	>25	32	0.39	2	0.07	SYN
*C. albicans* MYA-1003 (**G**)^b^	>25	32	0.39	4	0.15	SYN
POS						
*C. albicans* 10231 (**A**)^b^	0.62	32	0.15	8	0.5	SYN
*C. albicans* 64124 (**B**)^b^	>20	32	1.25	2	0.12	SYN
*C. albicans* MYA-2876 (**C**)^c^	10	16	1.25	1	0.18	SYN
*C. albicans* 90819 (**D**)^b^	>20	32	0.31	8	0.26	SYN
*C. albicans* MYA-2310 (**E**)^c^	10	16	0.62	1	0.12	SYN
*C. albicans* MYA-1237 (**F**)^b^	>20	32	0.31	2	0.07	SYN
*C. albicans* MYA-1003 (**G**)^b^	>20	32	1.25	2	0.12	SYN
VOR						
*C. albicans* 10231 (**A**)^b^	0.31	32	0.07	8	0.27	SYN
*C. albicans* 64124 (**B**)^b^	>10	32	1.25	16	0.62	IND
*C. albicans* MYA-2876 (**C**)^c^	>10	16	2.5	8	0.75	IND
*C. albicans* 90819 (**D**)^b^	>10	32	0.15	4	0.14	SYN
*C. albicans* MYA-2310 (**E**)^c^	10	16	0.15	1	0.07	SYN
*C. albicans* MYA-1237 (**F**)^b^	>10	32	0.31	2	0.09	SYN
*C. albicans* MYA-1003 (**G**)^b^	>10	32	0.31	4	0.14	SYN

^a^All of the strains are from ATCC.

^b^Indicates strains that are resistant to FLC, ITC, and VOR according to ATCC.

^c^Indicates strains that are susceptible to FLC, ITC, and VOR according to ATCC. *Note*: SYN indicates synergy (FICI ≤0.5) whereas IND indicates indifferent (FICI >0.5–4).

**Table 2:** *In vitro* susceptibility of yeast strains to **C**_**14**_ and azoles alone and in combination.

MICs of drugs (μg/mL)						
Drugs and Strains^a^	Alone		In combination		FICIs	Interpretation
	Azole	C_14_	Azole	C_14_		
FLC						
*C. albicans* 10231 (A)^b^	25	8	6.25	4	0.75	IND
*C. albicans* 64124 (B)^b^	>25	8	1.56	4	0.56	IND
*C. albicans* MYA-2876 (C)^c^	>25	8	12.5	4	1	IND
*C. albicans* 90819 (D)^b^	>25	8	6.25	2	0.5	SYN
*C. albicans* MYA-2310 (E)^c^	>25	8	6.25	2	0.5	SYN
*C. albicans* MYA-1237 (F)^b^	>25	8	0.78	2	0.28	SYN
*C. albicans* MYA-1003 (G)^b^	>25	8	12.5	4	1	IND
ITC						
*C. albicans* 10231 (A)^b^	0.78	8	0.19	2	0.5	SYN
*C. albicans* 64124 (B)^b^	>25	8	0.78	2	0.28	SYN
*C. albicans* MYA-2876 (C)^c^	12.5	8	1.56	4	0.62	IND
*C. albicans* 90819 (D)^b^	25	8	0.39	2	0.26	SYN
*C. albicans* MYA-2310 (E)^c^	12.5	8	0.78	1	0.18	SYN
*C. albicans* MYA-1237 (F)^b^	>25	8	3.12	1	0.25	SYN
*C. albicans* MYA-1003 (G)^b^	>25	8	0.78	2	0.28	SYN
POS						
*C. albicans* 10231 (A)^b^	0.62	8	0.15	2	0.49	SYN
*C. albicans* 64124 (B)^b^	>20	8	1.25	1	0.18	SYN
*C. albicans* MYA-2876 (C)^c^	10	8	1.25	2	0.37	SYN
*C. albicans* 90819 (D)^b^	>20	8	0.31	2	0.26	SYN
*C. albicans* MYA-2310 (E)^c^	10	8	0.31	2	0.28	SYN
*C. albicans* MYA-1237 (F)^b^	>20	8	1.25	1	0.18	SYN
*C. albicans* MYA-1003 (G)^b^	>20	8	1.25	2	0.31	SYN
VOR						
*C. albicans* 10231 (A)^b^	0.31	8	0.03	4	0.59	IND
*C. albicans* 64124 (B)^b^	>10	8	5	4	1	IND
*C. albicans* MYA-2876 (C)^c^	10	8	5	2	0.75	IND
*C. albicans* 90819 (D)^b^	>10	8	0.31	1	0.15	SYN
*C. albicans* MYA-2310 (E)^c^	10	8	0.15	1	0.14	SYN
*C. albicans* MYA-1237 (F)^b^	>10	8	0.31	1	0.15	SYN
*C. albicans* MYA-1003 (G)^b^	>10	8	2.5	1	0.37	SYN

^a^All of the strains are from ATCC.

^b^Indicates strains that are resistant to FLC, ITC, and VOR according to ATCC.

^c^Indicates strains that are susceptible to FLC, ITC, and VOR according to ATCC. *Note*: SYN indicates synergy (FICI ≤0.5) whereas IND indicates indifferent (FICI >0.5–4).
