# Supplementary material for: A combination approach to treating fungal infections
Source: Sci Rep. 2015 Nov 23;5:17070. doi: 10.1038/srep17070 (PMC4655404; doi:10.1038/srep17070)
Supplement: Supplementary Information [file srep17070-s1.pdf]

## Supporting Information

# A combination approach to treating fungal infections

*Sanjib K. Shrestha,*[a] *Marina Y. Fosso,*[a] *Sylvie Garneau-Tsodikova*[a,*]

[a] Department of Pharmaceutical Sciences, University of Kentucky, Lexington, KY, 40536-0596, USA.

(*Corresponding author: E-mail: sylviegtsodikova@uky.edu; Phone: 859-218-1686; Fax: 859-257-7585)

**Table S1.** Clinical sources and susceptibility/resistance profile of yeast strains against various antifungal agents according to ATCC reports.

| **Yeast strains**[a] | **Clinical sources** | **FLC** | **ITC** | **VOR** | **Caspofungin** |
|---|---|---|---|---|---|
| *C. albicans* 10231 (**A**) | Human (bronchomycosis) | R | R | R | S |
| *C. albicans* 64124 (**B**) | Human (mouth swab) | R | R | R | R |
| *C. albicans* MYA-2876 (**C**) | Human(clinical specimen) | S | S | S | S |
| *C. albicans* 90819 (**D**) | Human isolate | R | R | R | S |
| *C. albicans* MYA-2310 (**E**) | Grassland soil | S | S | S | S |
| *C. albicans* MYA-1237 (**F**) | Human (blood) | R | R | R | S |
| *C. albicans* MYA-1003 (**G**) | Human(Clinical isolate) | R | R | R | S |

[a] All of the strains are from ATCC. *Note*: R and S stand for resistant and sensitive, respectively. Strain **C** is the strain used for genome sequencing and Strain **E** is heterozygous for 5-FC resistance.

**Table S2.** Percentage of yeast cell growth using plate reader ($A_{600}$) used to determine MIC-2 (50% growth inhibition) values for FLC, ITC, POS, VOR, and caspofungin against *C. albicans* MYA-2876 (strain **C**) and *C. albicans* MYA-2310 (strain **E**).

| | *C. albicans* MYA-2876 (**C**) | | | | |
|---|---|---|---|---|---|
| **Concentration** (μg/mL) | **FLC** | **ITC** | **POS** | **VOR** | **Caspofungin** |
| **0.48** | 80% | 75% | 62% | 65% | 0% |
| **0.975** | 55% | 53% | 51% | 57% | 1% |
| **1.95** | 57% | 57% | 57% | 48% | 2% |
| **3.9** | 51% | 49% | 50% | 51% | 0% |
| **7.8** | 49% | 32% | 46% | 25% | 0% |
| **15.6** | 39% | 27% | 27% | 15% | 0% |
| **31.25** | 27% | 17% | 14% | 9% | 0% |
| | *C. albicans* MYA-2310 (**E**) | | | | |
| **Concentration** (μg/mL) | **FLC** | **ITC** | **POS** | **VOR** | **Caspofungin** |
| **0.48** | 87% | 84% | 70% | 56% | 4% |
| **0.975** | 84% | 87% | 54% | 62% | 3% |
| **1.95** | 55% | 50% | 45% | 54% | 3% |
| **3.9** | 51% | 67% | 50% | 48% | 3% |
| **7.8** | 49% | 56% | 51% | 44% | 7% |
| **15.6** | 55% | 49% | 32% | 21% | 8% |
| **31.25** | 54% | 33% | 29% | 16% | 3% |

**Table S3.** *In vitro* cytotoxicity of POS to $\mathbf{C_{12}}$ alone and in combination against A549 cell line.

| | [$\mathbf{C_{12}}$] (µg/mL) | | | | | | | | |
|---|---|---|---|---|---|---|---|---|---|
| [**POS**] (µg/mL) | **0** | **0.25** | **0.5** | **1** | **2** | **4** | **8** | **16** | **32** |
| **0** | 100 ± 16 | 94 ± 8 | 91± 3 | 100 ± 2 | 85 ± 21 | 100 ± 5 | 100 ± 6 | 90 ± 13 | 92 ± 11 |
| **0.31** | 93 ± 11 | 100 ± 14 | 100 ± 14 | 100 ± 12 | 100 ± 13 | 100 ± 9 | 100 ± 4 | 99 ± 1 | 89 ± 16 |
| **0.62** | 80 ± 8 | 100 ± 6 | 97 ± 4 | 100 ± 8 | 100 ± 14 | 100 ± 17 | 100 ± 6 | 89 ± 16 | 85 ± 15 |
| **1.25** | 68 ± 6 | 96 ± 20 | 92 ± 11 | 89 ± 19 | **100 ± 1**[a] | 94 ± 19 | 98 ± 12 | 82 ± 13 | 71 ± 18 |
| **2.5** | 58 ± 6 | 92 ± 14 | 96 ± 7 | 93 ± 10 | **100 ± 14**[b] | 90 ± 14 | 79 ± 6 | 80 ± 18 | 69 ± 5 |
| **5** | 49 ± 7 | 76 ± 3 | 71 ± 10 | 71 ± 6 | 89 ± 3 | 72 ± 2 | 70 ± 2 | 66 ± 1 | 62 ± 8 |
| **10** | 32 ± 12 | 41 ± 16 | 46 ± 4 | 42 ± 12 | 52 ± 4 | 45 ± 6 | 54 ± 5 | 47 ± 5 | 52 ± 4 |
| **20** | 7 ± 15 | 11 ± 10 | 18 ± 1 | 16 ± 8 | 20 ± 6 | 16 ± 8 | 29 ± 2 | 28 ± 1 | 22 ± 3 |

[a] The value in blue represents the combination of concentrations of $\mathbf{C_{12}}$ and POS used to determine FICI values against *C. albicans* ATCC 64124 (strain **B**) presented in Table 1. [b] The value in red represents the combination of concentrations of $\mathbf{C_{12}}$ and POS used to perform the time-kill curve studies presented in Fig. 2A.

**Table S4.** *In vitro* cytotoxicity of POS to $\mathbf{C_{12}}$ alone and in combination against BEAS-2B cell line.

| | [$\mathbf{C_{12}}$] (µg/mL) | | | | | | | | |
|---|---|---|---|---|---|---|---|---|---|
| [**POS**] (µg/mL) | **0** | **0.25** | **0.5** | **1** | **2** | **4** | **8** | **16** | **32** |
| **0** | 100 ± 8 | 100 ± 15 | 100 ± 8 | 100 ± 19 | 100 ± 7 | 100 ± 2 | 100 ± 5 | 100 ± 1 | 100 ± 1 |
| **0.31** | 84 ± 10 | 94 ± 9 | 100 ± 4 | 96 ± 6 | 100 ± 17 | 100 ± 18 | 100 ± 5 | 96 ± 17 | 84 ± 13 |
| **0.62** | 79 ± 2 | 100 ± 10 | 100 ± 9 | 88 ± 17 | 98 ± 1 | 98 ± 13 | 82 ± 1 | 77 ± 4 | 65 ± 2 |
| **1.25** | 79 ± 14 | 100 ± 16 | 98 ± 8 | 80 ± 9 | **90 ± 1**[a] | 86 ± 3 | 92 ± 11 | 72 ± 3 | 57 ± 3 |
| **2.5** | 75 ± 17 | 99 ± 8 | 90 ± 3 | 88 ± 8 | **81 ± 7**[b] | 76 ± 19 | 70 ± 20 | 61 ± 2 | 49 ± 6 |
| **5** | 71 ± 5 | 95 ± 2 | 86 ± 4 | 80 ± 3 | 79 ± 9 | 73 ± 16 | 65 ± 28 | 52 ± 12 | 46 ± 4 |
| **10** | 53 ± 5 | 63 ± 9 | 58 ± 3 | 45 ± 8 | 61 ± 10 | 66 ± 4 | 55 ± 20 | 54 ± 7 | 42 ± 1 |
| **20** | 20 ± 6 | 21 ± 1 | 22 ± 1 | 20 ± 2 | 25 ± 3 | 24 ± 1 | 20 ± 6 | 22 ± 7 | 21 ± 11 |

[a] The value in blue represents the combination of concentrations of $\mathbf{C_{12}}$ and POS used to determine FICI values against *C. albicans* ATCC 64124 (strain **B**) presented in Table 1. [b] The value in red represents the combination of concentrations of $\mathbf{C_{12}}$ and POS used to perform the time-kill curve studies presented in Fig. 2A.

**Table S5.** *In vitro* cytotoxicity of POS to $\mathbf{C_{14}}$ alone and in combination against A549 cell line.

| | [$\mathbf{C_{14}}$] (µg/mL) | | | | | | | | |
|---|---|---|---|---|---|---|---|---|---|
| [**POS**] (µg/mL) | **0** | **0.06** | **0.12** | **0.25** | **0.5** | **1** | **2** | **4** | **8** |
| **0** | 100 ± 5 | 100 ± 3 | 100 ± 9 | 100 ± 2 | 100 ± 3 | 100 ± 21 | 100 ± 1 | 100 ± 6 | 100 ± 16 |
| **0.31** | 87 ± 18 | 100 ± 4 | 100 ± 4 | 100 ± 12 | 100 ± 1 | 100 ± 4 | 100 ± 7 | 100 ± 4 | 100 ± 16 |
| **0.62** | 91 ± 13 | 100 ± 11 | 100 ± 9 | 100 ± 7 | 100 ± 4 | 100 ± 1 | 100 ± 9 | 100 ± 28 | 92 ± 6 |
| **1.25** | 88 ± 16 | 100 ± 8 | 100 ± 5 | 95 ± 10 | 100 ± 15 | **100 ± 8**[a] | **100 ± 1**[b] | 89 ± 4 | 74 ± 12 |
| **2.5** | 94 ± 9 | 100 ± 2 | 100 ± 6 | 100 ± 2 | 100 ± 2 | 98 ± 3 | **85 ± 3**[a] | 85 ± 11 | 76 ± 14 |
| **5** | 92 ± 11 | 82 ± 6 | 76 ± 3 | 78 ± 15 | 98 ± 9 | 78 ± 6 | 76 ± 7 | 71 ± 6 | 65 ± 2 |
| **10** | 77 ± 11 | 44 ± 20 | 44 ± 2 | 45 ± 16 | 55 ± 9 | 47 ± 4 | 57 ± 1 | 49 ± 3 | 55 ± 9 |
| **20** | 37 ± 6 | 9 ± 6 | 15 ± 3 | 14 ± 5 | 19 ± 4 | 14 ± 5 | 27 ± 3 | 27 ± 1 | 20 ± 1 |

[a] The values in blue represent the combinations of concentrations of $\mathbf{C_{14}}$ and POS used to determine FICI values against *C. albicans* ATCC 64124 (strain **B**) presented in Table 2. [b] The value in red represents the combination of concentrations of $\mathbf{C_{14}}$ and POS used to perform the time kill-curve studies presented in Fig. 2B.

**Table S6.** *In vitro* cytotoxicity of POS to $\mathbf{C_{14}}$ alone and in combination against BEAS-2B cell line.

| [**POS**] (μg/mL) | [$\mathbf{C_{14}}$] (μg/mL) | | | | | | | | |
|---|---|---|---|---|---|---|---|---|---|
| | **0** | **0.06** | **0.12** | **0.25** | **0.5** | **1** | **2** | **4** | **8** |
| **0** | 100 ± 5 | 78 ± 6 | 79 ± 3 | 87 ± 14 | 78 ± 3 | 90 ± 1 | 100 ± 2 | 82 ± 9 | 78 ± 1 |
| **0.31** | 75 ± 10 | 85 ± 17 | 92 ± 17 | 100 ± 4 | 95 ± 18 | 100 ± 5 | 100 ± 5 | 81 ± 21 | 80 ± 14 |
| **0.62** | 75 ± 7 | 78 ± 7 | 92 ± 19 | 98 ± 3 | 100 ± 10 | 100 ± 1 | 100 ± 5 | 96 ± 4 | 81 ± 5 |
| **1.25** | 65 ± 1 | 86 ± 7 | 88 ± 20 | 92 ± 1 | 100 ± 1 | **100 ± 5**[a] | **100 ± 1**[b] | 95 ± 10 | 81 ± 8 |
| **2.5** | 64 ± 5 | 100 ± 7 | 98 ± 11 | 100 ± 3 | 100 ± 3 | 100 ± 2 | **100 ± 5**[a] | 93 ± 12 | 84 ± 1 |
| **5** | 64 ± 9 | 100 ± 2 | 100 ± 11 | 100 ± 2 | 100 ± 3 | 100 ± 8 | 100 ± 4 | 100 ± 3 | 100 ± 6 |
| **10** | 60 ± 7 | 59 ± 9 | 72 ± 16 | 72 ± 16 | 88 ± 13 | 84 ± 5 | 83 ± 6 | 74 ± 8 | 70 ± 5 |
| **20** | 13 ± 2 | 10 ± 3 | 15 ± 3 | 24 ± 2 | 36 ± 6 | 29 ± 9 | 34 ± 12 | 24 ± 8 | 23 ± 1 |

[a] The values in blue represent the combinations of concentrations of $\mathbf{C_{14}}$ and POS used to determine FICI values against *C. albicans* ATCC 64124 (strain **B**) presented in Table 2. [b] The value in red represents the combination of concentrations of $\mathbf{C_{14}}$ and POS used to perform the time-kill curve studies presented in Fig. 2B.
